# Comparison of Ritchie and Kato–Katz methods for the detection of intestinal helminths in humans: a systematic review and meta-analysis

**DOI:** 10.1186/s13071-026-07437-7

**Published:** 2026-05-15

**Authors:** Jurairat Jongthawin, Aongart Mahittikorn, Kinley Wangdi, Frederick Ramirez Masangkay, Manas Kotepui

**Affiliations:** 1 Faculty of Medicine, Mahasarakham University, Maha Sarakham, 44000 Thailand; 2 Department of Protozoology, Faculty of Tropical Medicine, Mahidol University, Bangkok, 10400 Thailand; 3 HEAL Global Research Center, Health Research Institute, Faculty of Health, University of Canberra, Bruce, ACT 2617 Australia; 4 National Centre for Epidemiology and Population Health, College of Law, Governance and Policy, Australian National University, Acton, ACT 2601 Australia; 5 Department of Medical Technology, Faculty of Pharmacy, University of Santo Tomas, 1008 Manila, Philippines; 6 Research Center for the Natural and Applied Sciences, University of Santo Tomas, 1008 Manila, Philippines; 7 Medical Technology Program, Faculty of Science, Nakhon Phanom University, Nakhon Phanom, 48000 Thailand

**Keywords:** Soil-transmitted helminth, Soil-transmitted helminthiasis, Formalin–ether concentration technique, Formalin-ethyl acetate concentration technique, FEC, Kato–Katz, Kato, Systematic review, Meta-analysis

## Abstract

**Background:**

Comparative evidence on the diagnostic performance of Formalin-Ether Concentration (FEC) versus the Kato–Katz thick smear (KK) for detecting intestinal helminths in humans remains inconsistent. This systematic review and meta-analysis compared the odds of detecting intestinal helminths using FEC versus the single-slide KK.

**Methods:**

A systematic search was conducted across six databases (PubMed, Scopus, EMBASE, Web of Science, Nursing and Allied Health Premium, and Ovid). Eligible studies directly compared FEC with single-slide KK for stool examination. Data was extracted using a standardized template, and risk of bias was assessed using the Joanna Briggs Institute (JBI) tools. Pooled odds ratios (ORs) were estimated using fixed-effect and random-effects models. Heterogeneity (*I*^*2*^), subgroup analyses, meta-regression, and assessments of publication bias (funnel plots, Harbord tests, and trim-and-fill) were performed where appropriate.

**Results:**

A total of 40 studies met the inclusion criteria. For overall intestinal helminths (27 studies; 11,198 samples), the random-effects model produced a nonsignificant pooled OR of 0.76 (95% confidence interval [CI]: 0.55–1.05, *I*^*2*^ = 94.2%), whereas the fixed-effect model indicated higher detection by KK (OR = 0.84; 95% CI 0.78–0.91). For soil-transmitted helminth infections (STHs) (12 studies; 3615 samples), pooled estimates were 0.98 (95% CI 0.61–1.55, *I*^*2*^ = 71.3%; random-effects) and 0.84 (95% CI 0.74–0.97; fixed-effect). Species-specific pooled ORs were nonsignificant for hookworm (0.75; 95% CI 0.48–1.18, *I*^*2*^ = 83.2%), *Ascaris lumbricoides* (0.94; 95% CI 0.63–1.40, *I*^*2*^ = 80.8%), and *Trichuris trichiura* (0.65; 95% CI 0.40–1.07, *I*^*2*^ = 68.2%). FEC detected *Strongyloides stercoralis* significantly more frequently (OR = 5.02; 95% CI 1.75–14.41, *I*^*2*^ = 0%), while KK detected *Schistosoma mansoni* more often (OR = 0.59; 95% CI 0.39–0.90,* I*^*2*^ = 93.7%). For *Opisthorchis viverrini*, no difference was observed between the two detection methods (OR = 1.09; 95% CI 0.61–1.92,* I*^*2*^ = 86.7%). Subgroup analyses revealed notable regional and species-level variation. Meta-regression indicated minimal influence of study design, continent, or participant type. Publication bias assessments suggested minimal small-study effects.

**Conclusions:**

The diagnostic performance varied substantially between FEC methods and KK, depending on helminth species, region, and study design. KK was more sensitive at detecting *S. mansoni* and often performed similarly to or better than other methods for STHs, particularly in low-intensity settings. In contrast, FEC methods showed superior detection of *S. stercoralis* and preserved hookworm eggs. These findings support the use of integrated diagnostic strategies that combine KK with sedimentation or molecular methods to enhance surveillance in the era of helminth control and elimination.

**Graphical Abstract:**

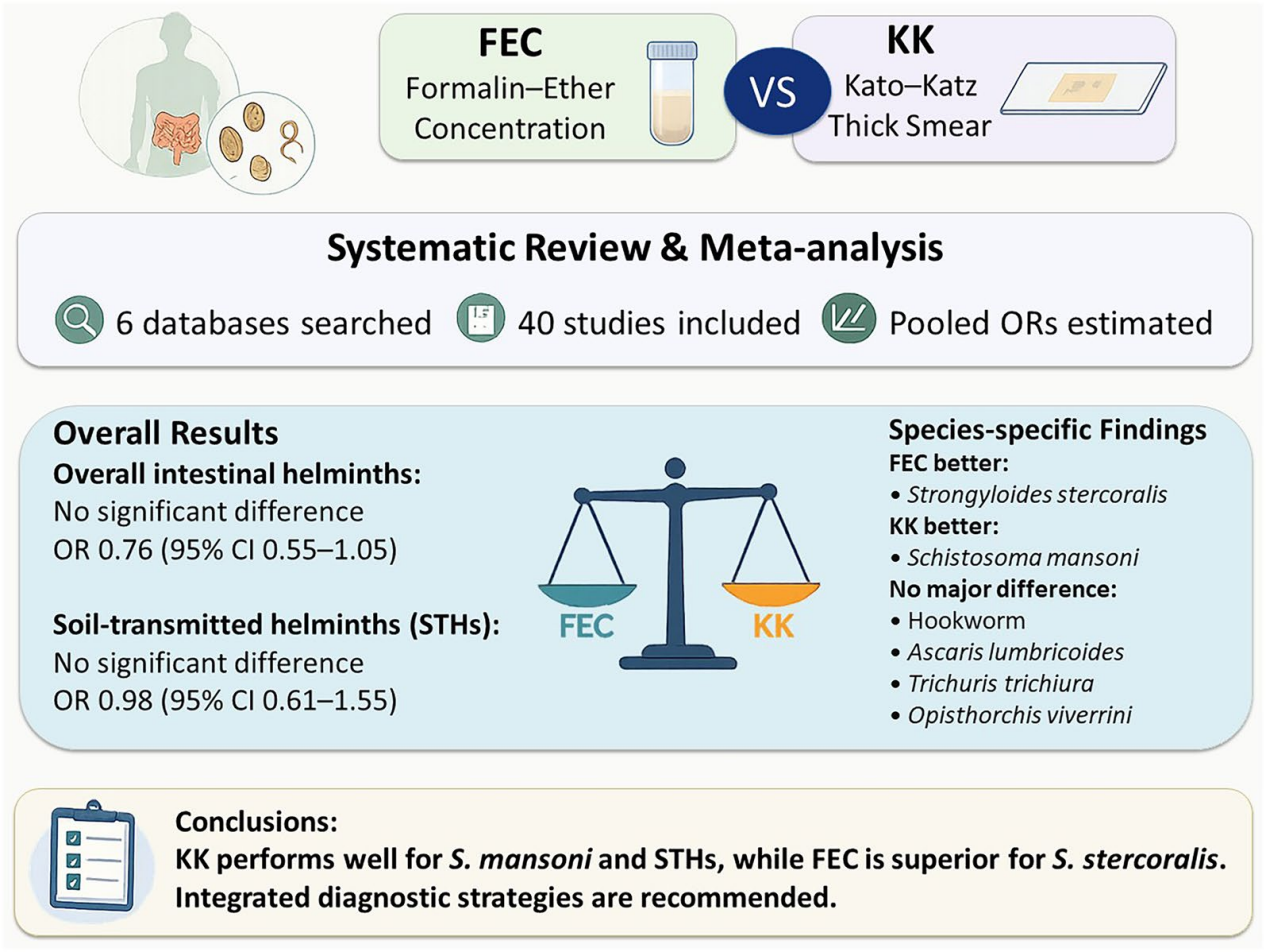

**Supplementary Information:**

The online version contains supplementary material available at 10.1186/s13071-026-07437-7.

## Background

Soil-transmitted helminth (STH) infections and schistosomiasis remain among the most prevalent neglected tropical diseases (NTDs) worldwide. It is estimated that approximately 1.5 billion people are infected with STHs (*Ascaris lumbricoides*, *Trichuris trichiura*, and hookworms) and over 200 million with *Schistosoma* species, primarily in tropical and subtropical regions with limited access to clean water and sanitation [1, 2]. These infections contribute significantly to the global burden of disease, causing anemia, malnutrition, physical growth retardation, and cognitive impairment, particularly in school-aged children and women of reproductive age [3]. Global control strategies, driven largely by the World Health Organization (WHO), rely on preventive chemotherapy (PC) through mass drug administration (MDA) to reduce morbidity [4]. As these programs succeed and infection intensities decline, the focus in many regions is shifting from morbidity control towards the interruption of transmission and elimination [5].

Accurate diagnostic tools are crucial for monitoring and evaluating these control programs. The reliability of prevalence data dictates the frequency of MDA and the decision to stop interventions. Currently, the WHO recommends the Kato-Katz thick smear (KK) as the standard diagnostic method for epidemiological surveys of intestinal helminths and *Schistosoma mansoni* owing to its simplicity, low cost, and ability to provide quantitative data (eggs per gram of stool) [6]. However, the limitations of KK are well-documented. Its sensitivity is heavily influenced by day-to-day variations in egg excretion and is notably reduced in low-intensity infections, a scenario increasingly common in areas under successful PC coverage [7, 8]. Furthermore, the rapid clearing of delicate hookworm eggs in KK preparations (often within 30–60 min) poses a significant logistical challenge in large-scale field surveys, potentially leading to underestimations of hookworm prevalence [7, 9, 10].

Conversely, formalin-based sedimentation methods, such as the Formalin–Ether Concentration (FEC), as originally described by Ritchie [11] and later modified to substitute ether for ethyl-acetate [12], have been widely used in clinical laboratories. These methods are valued for their ability to concentrate a larger volume of stool, clear debris, and improve the detection of a broader spectrum of parasites, including protozoa and helminth larvae [13]. Additionally, the use of a fixative prevents the degradation of hookworm eggs, allowing for delayed examination, which is operationally advantageous in field settings [10, 14].

Despite the emergence of more advanced diagnostic approaches, such as molecular and antigen-based assays, their application in many endemic settings remains limited owing to high costs, specialized equipment requirements, and the need for technical expertise. As a result, conventional microscopy-based methods, including KK and FEC methods, remain the most widely used diagnostic tools in large-scale surveillance programs, routine clinical practice, and field-based studies in low- and middle-income countries. Therefore, understanding the comparative performance of these commonly used methods remains highly relevant for informing diagnostic strategies in resource-constrained settings.

Comparative studies have yielded varying results regarding the relative performance of FEC versus KK. While some studies suggest that FEC is more sensitive or more accurate than KK in detecting intestinal parasite infections [15–19], other reports indicate that KK may still be more sensitive for *Schistosoma mansoni* owing to the larger amount of stool matrix examined in a thick smear compared with the final sediment of FEC [20–23]. These inconsistencies highlight the need for a comprehensive synthesis of available evidence to guide the selection of appropriate diagnostic methods in different epidemiological and operational contexts. This gap limits the ability of national programs and health facilities to choose the most appropriate diagnostic approach on the basis of local epidemiological needs and operational constraints. To address these gaps, a systematic review and meta-analysis have been conducted to compare the odds of detecting intestinal helminths using FEC methods with those of the single-slide KK across different helminth species. This systematic review and meta-analysis will provide an evidence-based assessment to inform diagnostic selection in surveillance, research, and routine clinical practice.

## Methods

### Registration and protocol

This systematic review and meta-analysis were conducted in accordance with the PRISMA 2020 guidelines [24]. The protocol was prospectively registered in PROSPERO (CRD420251164374).

### Systematic review framework

The systematic review was structured according to the Population, Exposure, Comparator, and Outcomes (PECO) framework [25]. Population (P) consists of human participants of any age undergoing stool examination for intestinal helminth infections, including STH such as *A. lumbricoides*, *T. trichiura*, hookworm species, and *S. stercoralis*, as well as non-STH infections such as *S. mansoni* and *O. viverrini*. Exposure (E) is stool examination using the FEC method or its modified equivalents (e.g., formalin–ethyl acetate concentration), or other validated biphasic sedimentation methods (single slide). The comparator (C) is stool examination using the KK thick smear technique (single slide). Outcomes (O) include: (i) the primary outcome, which is the odds ratio (OR) of detecting intestinal helminth (STH infections) comparing FEC with KK, and (ii) secondary outcomes, which are the ORs for detecting non-STH helminths comparing FEC with KK.

### Searches

A systematic search was conducted across six electronic databases: PubMed, Scopus, EMBASE, Web of Science, Nursing and Allied Health Premium, and Ovid from inception to January 2025. Search terms combined controlled vocabulary and free-text keywords related to intestinal helminths, STHs, diagnostic methods, FEC, and KK. The general search strategy is “(“Thick smear” OR “Kato-Katz” OR “Kato smear” OR “Kato’s thick smear” OR “Kato-thick smear”) AND (“Formol–ether Concentration” OR “Formol ether sedimentation” OR “Formalin–ether Concentration” OR “Formalin ether Concentration” OR “Formalin ethyl acetate concentration” OR “Formalin–ethyl acetate concentration” OR “Formalin ethyl acetate sedimentation” OR “Formalin–ethyl acetate sedimentation”).” No language restrictions were applied. Full search strategies for each database are provided in Supplementary Supplementary Table S1.

### Eligibility criteria

Studies were eligible if they evaluated stool-based detection of intestinal helminths, included a comparison between FEC (including modifications such as formalin–ethyl acetate concentration) and KK. Cross-sectional studies and diagnostic accuracy studies were included. Reviews, conference abstracts, correspondence, studies lacking a valid comparison, or those not performing either KK or FEC were excluded.

### Study selection

All retrieved records were imported into EndNote (version 21.0, Philadelphia, PA, USA) for deduplication. Screening was conducted in two stages: title/abstract screening followed by full-text review. Two authors (M.K., A.M.) independently assessed eligibility and resolved disagreements through discussion. Reasons for exclusion at each stage were recorded.

### Data extraction

Data extraction using a standardized form was conducted by one author (M.K.) and cross-checked by another author (J.J.) for accuracy. Any disagreement was resolved through discussion to create a consensus. Extracted information included study characteristics (year, country, design, population), diagnostic methods evaluated, sample size, and the number of positive detections per method.

### Risk of bias assessment

The quality of included studies was evaluated using the Joanna Briggs Institute (JBI) critical appraisal tools for cross-sectional and diagnostic accuracy studies [26]. Two authors (M.K., J.J.) made judgments independently.

### Statistical analysis

The principal effect measure was the odds ratios (OR) comparing detection by FEC with detection by KK. Meta-analyses were performed using both fixed-effect and random-effects models (DerSimonian–Laird) [27]. Statistical heterogeneity was assessed using *I*^*2*^ statistics, with *I*^*2*^ of 25%, 50%, and 75% indicating low, moderate, and high heterogeneity, respectively [28]. When 10 or more studies were available, subgroup analyses and meta-regression examined whether study design, continent, country, or participant type explained variation in ORs. Funnel plots, Harbord tests, and trim-and-fill procedures were used to assess small-study effects and potential publication bias. For outcomes with fewer than 10 studies, meta-regression and formal tests for funnel plot asymmetry were not performed owing to limited statistical power [29]. All analyses were conducted using RStudio (Version 2024.04.2 + 764) [30]. A significance threshold of *P* < 0.05 was applied throughout the study.

## Results

### Search results

**Fig. 1:**
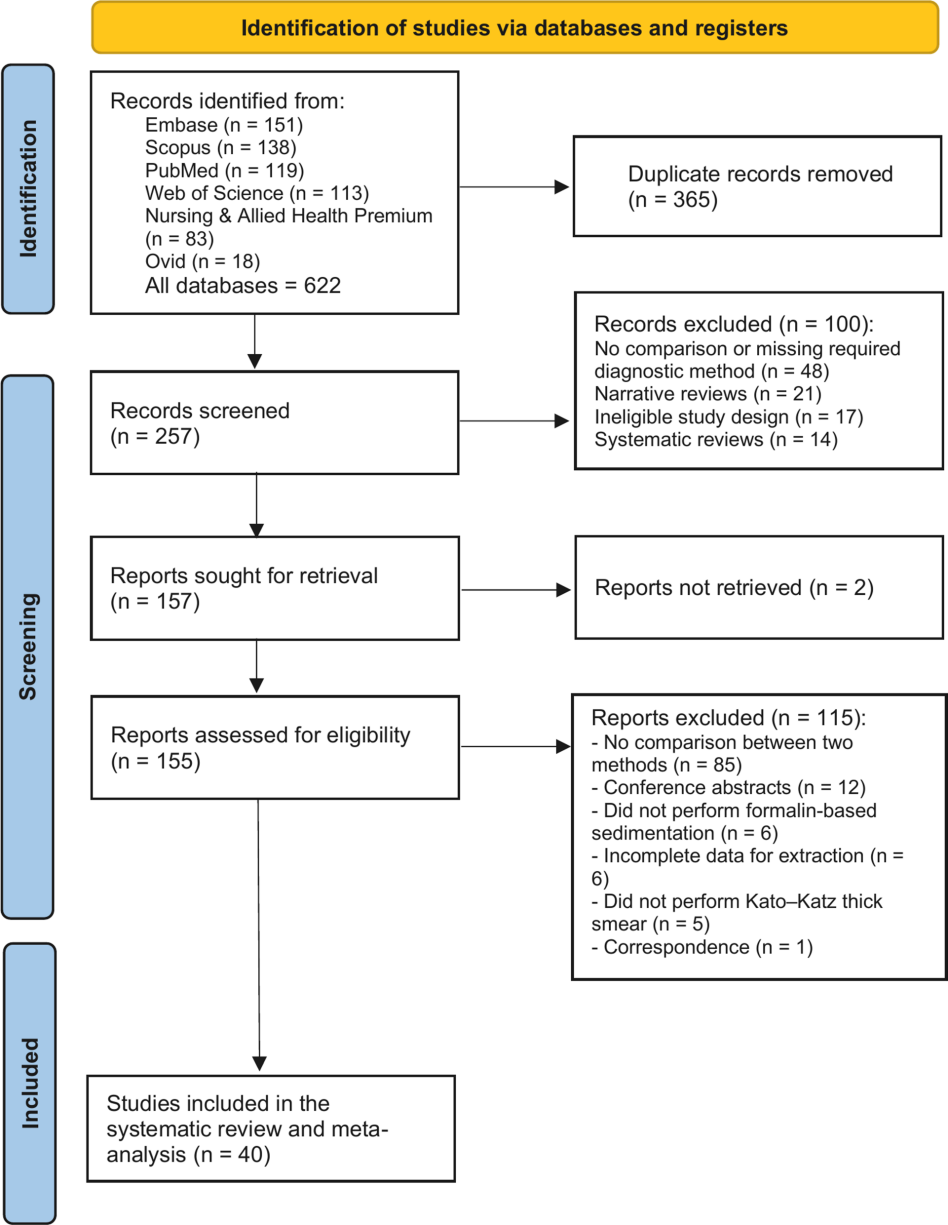
The PRISMA flow diagram summarizes the study selection process for the systematic review

A total of 622 records were identified across six databases. After removing 365 duplicates, 257 records remained for screening. Of these, 100 records were excluded based on title and abstract, owing to a lack of comparison between diagnostic methods, narrative or systematic reviews, or an ineligible study design. The remaining 157 reports were sought for retrieval; however, two reports could not be obtained. A total of 155 full-text reports were assessed for eligibility, and 115 were excluded for reasons such as the lack of a comparison between diagnostic methods, conference abstracts, failure to perform FEC or KK, incomplete extractable data, and being correspondence. Finally, 40 studies met the inclusion criteria and were included in the systematic review and meta-analysis (Fig. 1).

### Characteristics of included studies

A total of 40 studies published between 1996 and 2025 met the inclusion criteria. Most studies (34/40; 85%) employed a cross-sectional design, while six studies (15%) were diagnostic accuracy investigations. The studies were conducted across Asia (16 studies; 40%), Africa (22 studies; 55%), and South America (2 studies; 5%), with Ethiopia and Thailand being the most frequently studied countries. Study populations included community members (37.5%), schoolchildren (37.5%), attendees at health facilities, preschool-aged children, pregnant women, and migrant workers. Regarding diagnostic methodology, formol/formalin ether concentration (31 studies; 77.5%), with several studies employing modified or alternative sedimentation methods (Table 1, Supplementary Table S2).

**Table 1:** Characteristics of included studies

Characteristics	Category	No. (%)	Refs.
Publication years	1996–2025	40 (100)	[10, 15–17, 21, 23, 31–64]
Study design	Cross-sectional study	34 (85%)	[15–17, 21, 23, 31–59]
	Diagnostic accuracy study	6 (15%)	[10, 60–64]
Study location	Asia	16 (40%)	
	Thailand	5 (12.5%)	[17, 39, 46, 50, 62]
	Lao PDR	4 (10%)	[48, 52, 53, 55]
	Cambodia	2 (5%)	[38, 54]
	Philippines	2 (5%)	[33, 37]
	China	1 (2.5%)	[64]
	Indonesia	1 (2.5%)	[63]
	Bangladesh	1 (2.5%)	[49]
	Africa	22 (55%)	
	Ethiopia	12 (30%)	[15, 31, 32, 34, 36, 42–44, 47, 56, 58, 59]
	Egypt	6 (15%)	[16, 21, 23, 35, 45, 60]
	Burkina Faso	1 (2.5%)	[40]
	Cameroon	1 (2.5%)	[57]
	Malawi	1 (2.5%)	[10]
	Mali	1 (2.5%)	[41]
	South America	2 (5%)	
	Ecuador	2 (5%)	[51, 61]
Types of participants	Participants in the community	15 (37.5%)	[17, 23, 34, 39, 41, 42, 48, 50–52, 54, 55, 61, 62, 64]
	Schoolchildren	15 (37.5%)	[15, 31, 33, 36–38, 42–45, 47, 56, 58, 60, 63]
	Participants who came to health institutions	4 (10%)	[16, 32, 35, 59]
	Participants who came to health institutions and participants in the community	1 (2.5%)	[53]
	Schoolchildren and adults	1 (2.5%)	[49]
	Pregnant women	1 (2.5%)	[57]
	Preschool-aged children	1 (2.5%)	[40]
	Migrant workers	1 (2.5%)	[46]
	Not specified	1 (2.5%)	[10]
Detection technique	Formol/formalin ether concentration	31 (77.5%)	[35, 37, 38, 41, 48, 49, 60, 61, 64]
	Formalin–ethyl acetate concentration	6 (15%)	[17, 39, 52, 53, 55, 62]
	Modified formalin ether concentration	1 (2.5%)	[46]
	One-step Formalin–ethyl acetate concentration, conventional FEC	1 (2.5%)	[50]
	Sodium acetate acetic acid formalin (SAF) for formalin ether concentration	1 (2.5%)	[31]

### Risk of bias

Among the 34 cross-sectional studies, 29 (85.3%) were assessed as having a low risk of bias, while 11 (27.5%) were assessed as having a moderate risk of bias. No study was classified as high risk. Among six diagnostic test accuracy studies, all studies were assessed as having a low risk of bias (Supplementary Table S3).

### Comparison of diagnostic methods for intestinal helminth infections

**Fig. 2:**
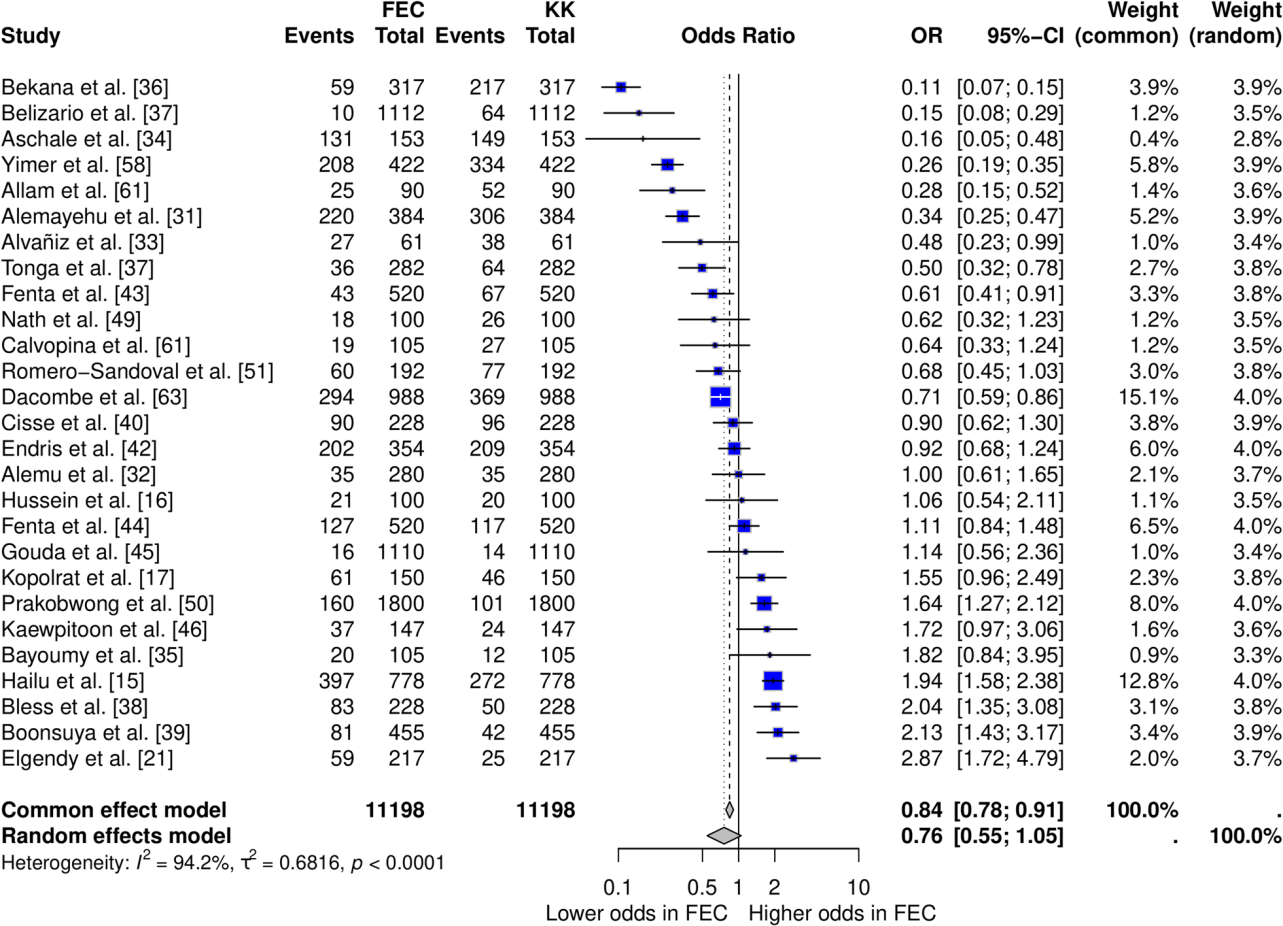
The forest plot shows the pooled odds ratio for intestinal helminth infections comparing FEC with KK. Each horizontal line represents an individual study with its corresponding odds ratio (OR) and 95% confidence interval (CI). The blue squares indicate the point estimates of the ORs, and their sizes are proportional to the weight assigned to each study in the meta-analysis. The diamond at the bottom represents the pooled effect estimate for both the common (fixed) and random-effects model

The meta-analysis on the OR for intestinal helminth infections included 27 studies, totaling 11,198 stool samples per diagnostic method. The random-effects model yielded a nonsignificant pooled OR of 0.76 (95% confidence interval [CI]: 0.55–1.05, *P* = 0.098, *I*^*2*^ = 94.2%), while the fixed-effect model estimated a statistically significant OR of 0.84 (95% CI 0.78–0.91, *P* < 0.0001, Fig. 2).

The meta-regression indicated that the study design, country, continent, and participant type did not significantly influence the pooled OR (*P* > 0.05, Supplementary Table S4). However, the subgroup analysis showed variations across these factors (Supplementary Table S4). When stratified by study design, diagnostic accuracy studies showed a significantly lower OR (0.53, 95% CI 0.30–0.92) than cross-sectional studies (OR = 0.80, 95% CI 0.56–1.14). Regional variation was noted. Thailand and Cambodia reported higher detection by concentration methods (OR > 1), while Ethiopia, the Philippines, and South America showed lower OR (< 1). By participant type, schoolchildren had a significantly lower OR (0.56, 95% CI 0.34–0.94). In contrast, studies involving community participants, hospital patients, and migrant workers showed ORs closer to or above 1.

The funnel plot for the comparison of FEC versus KK in detecting helminth infections appears largely symmetrical (Supplementary Fig. 1), and the statistical test for funnel plot asymmetry (Harbord test) shows no evidence of publication bias (*t* = −0.48, *P* = 0.6365). This indicates that small-study effects are unlikely to be influencing the results. The trim-and-fill method estimated that three studies might be missing, but after adjusting for these, the pooled OR (OR = 0.93, 95% CI 0.64–1.35) remained similar to the original estimate and nonsignificant.

### Comparison of diagnostic methods for STH infections

**Fig. 3:**
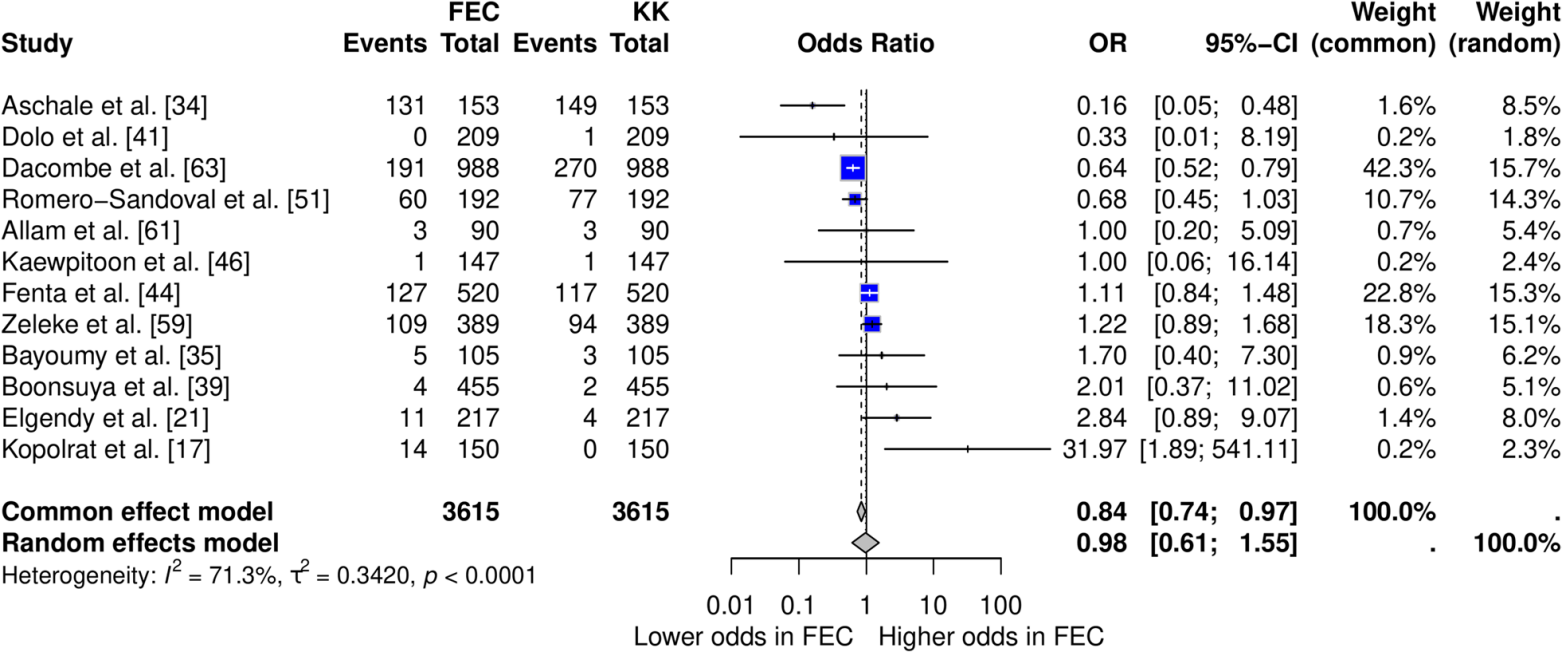
The Forest plot shows the pooled odds ratio for STH infections comparing FEC with KK. Each horizontal line represents an individual study with its corresponding odds ratio (OR) and 95% confidence interval (CI). The blue squares indicate the point estimates of the ORs, and their sizes are proportional to the weight assigned to each study in the meta-analysis. The diamond at the bottom represents the pooled effect estimate for both the common (fixed) and random-effects model

The meta-analysis on the OR of STH infections included 12 studies, totaling 3615 stool samples per diagnostic method. The random-effects model yielded a nonsignificant pooled OR of 0.98 (95% CI 0.61–1.55, *P* = 0.92, *I*^*2*^ = 71.3%), while the fixed-effect model estimated a statistically significant OR of 0.84 (95% CI 0.74–0.97, *P* = 0.02; Fig. 3).

The meta-regression analysis revealed that the study design, country, continent, and participant type did not significantly influence the pooled proportion ratio for STH infections between concentration methods and KK (*P* > 0.05). Across subgroups, diagnostic accuracy studies showed a lower pooled OR (OR = 0.64, 95% CI 0.52–0.79) compared with cross-sectional studies. Regional differences were noted, with slightly higher ratios reported in Asia (OR = 3.46, 95% CI 0.59–20.3) and lower ratios in Africa (OR = 0.89, 95% CI 0.51–1.57). Among participant types, the diagnostic performance varied across groups; however, participants from the community, schoolchildren, and patients from hospitals or health institutions showed no significant difference between the two diagnostic methods (Supplementary Table S5).

The funnel plot for the comparison of FEC versus KK in detecting STH infections appears largely symmetrical (Supplementary Fig. 2), and the statistical test for funnel plot asymmetry (Harbord test) shows no evidence of publication bias (*t* = 0.86, *P* = 0.4085). This indicates that small-study effects are unlikely to be influencing the pooled estimate. The trim-and-fill analysis suggested that two studies might be missing, but after imputing these studies, the adjusted pooled OR (0.81; 95% CI 0.48–1.39) remained nonsignificant and similar to the original estimate.

### Comparison of diagnostic methods for hookworm infections

**Fig. 4:**
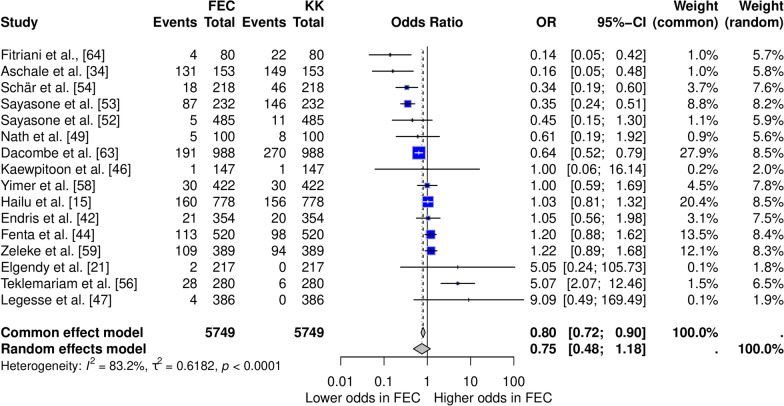
The Forest plot shows the pooled odds ratio for hookworm infections comparing FEC with KK. Each horizontal line represents an individual study with its corresponding odds ratio (OR) and 95% confidence interval (CI). The blue squares indicate the point estimates of the ORs, and their sizes are proportional to the weight assigned to each study in the meta-analysis. The diamond at the bottom represents the pooled effect estimate for both the common (fixed) and random-effects model

The meta-analysis of the OR for hookworm infections included 16 studies, totaling 5749 stool samples per diagnostic method. The random-effects model yielded a non-significant pooled OR of 0.75 (95% CI 0.48–1.18, *P* = 0.098, *I*^*2*^ = 83.2%), while the fixed-effect model estimated a statistically significant OR of 0.80 (95% CI 0.72–0.90, *P* < 0.0001, Fig. 4).

The meta-regression analysis indicated that none of the examined moderators: study design, country, continent, or participant type, significantly explained the heterogeneity in the proportion ratio of hookworm detection between FEC and the KK (*P* > 0.05 for all). When stratified by continent, concentration methods showed significantly lower detection rates in Asia (OR = 0.35, 95% CI 0.26–0.46) compared with Africa (OR = 1.10, 95% CI 0.66–1.81). Among participant groups, community-based studies showed a lower OR (0.33, 95% CI 0.21–0.52), whereas studies in schoolchildren and hospital patients demonstrated higher but more variable estimates (Supplementary Table S5).

The funnel plot appears symmetrical (Supplementary Fig. 3), suggesting no clear visual evidence of publication bias. This is supported by the Harbord regression test for funnel plot asymmetry (*t* = 0.05, *P* = 0.9624), which shows statistically nonsignificant small-study effects. The trim-and-fill analysis did not impute any missing studies, further indicating the absence of meaningful publication bias. The pooled random-effects OR remained nonsignificant (OR = 0.75; 95% CI 0.48–1.18), confirming that adjusting for potential bias does not change the overall conclusion.

### Comparison of diagnostic methods for *A. lumbricoides* infections

**Fig. 5:**
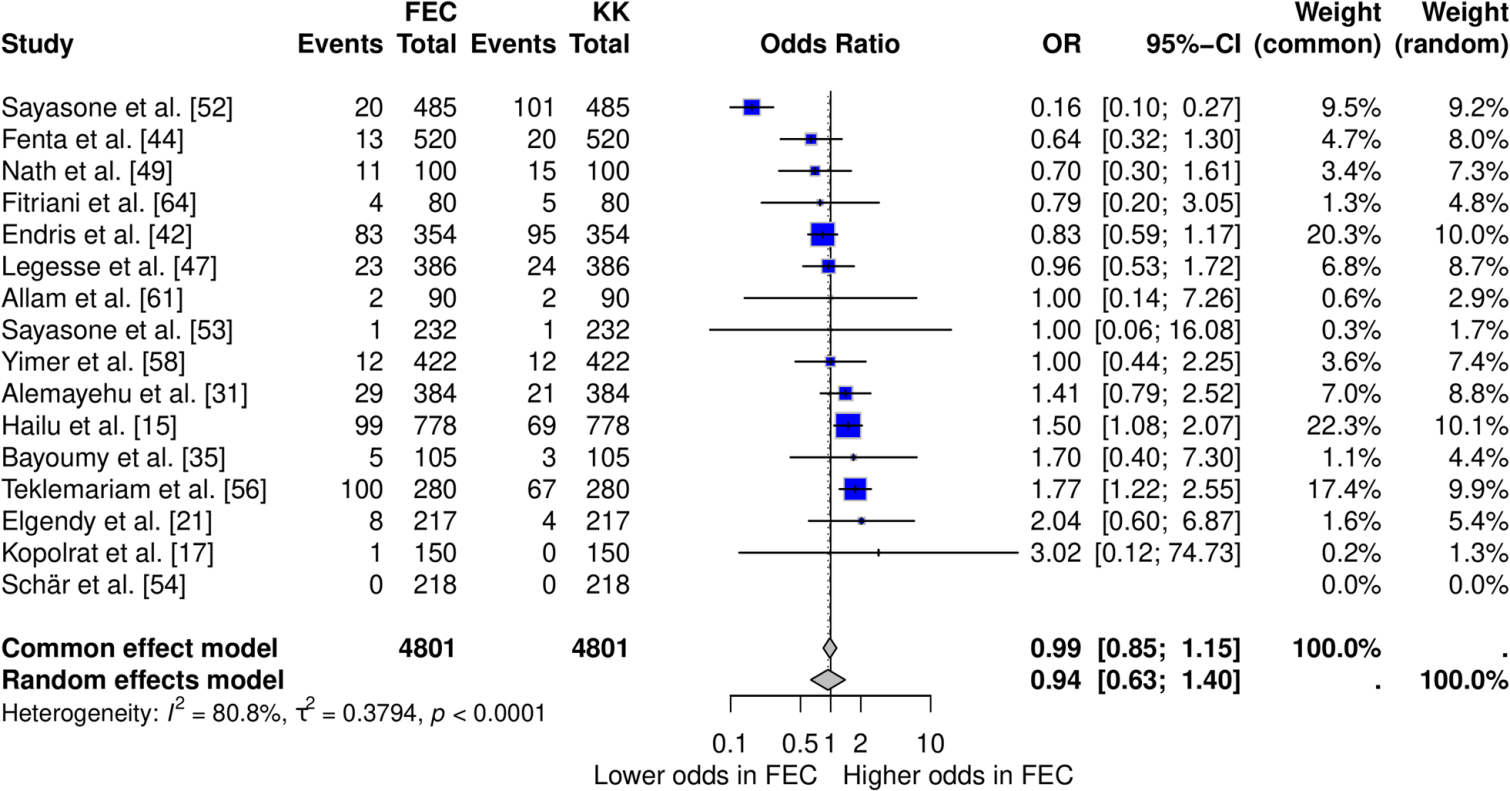
The Forest plot shows the pooled odds ratio for *Ascaris lumbricoides* infections comparing FEC with KK. Each horizontal line represents an individual study with its corresponding odds ratio (OR) and 95% confidence interval (CI). The blue squares indicate the point estimates of the ORs, and their sizes are proportional to the weight assigned to each study in the meta-analysis. The diamond at the bottom represents the pooled effect estimate for both the common (fixed) and random-effects model

The meta-analysis of the OR for *A. lumbricoides* infections included 16 studies comprising a total of 4801 stool samples across two diagnostic methods. Both the random-effects and fixed-effect models yielded nonsignificant pooled ORs of 0.94 (95% CI 0.63–1.40, *P* = 0.77, *I*^*2*^ = 80.8%) and 0.99 (95% CI 0.85–1.15, *P* = 0.86, Fig. 5), respectively.

The meta-regression analysis indicated that the country (*P* = 0.0001) and continent (*P* = 0.0023) were significant moderators influencing the proportion ratio of *A. lumbricoides* detection between FEC and KK, whereas study design and participant type were not significant (*P* > 0.05). Studies conducted in Africa reported higher detection rates with concentration methods (OR = 1.19, 95% CI 0.92–1.54), whereas those in Asia showed lower ratios (OR = 0.51, 95% CI 0.19–1.34). Among participant groups, no significant differences were found; however, school-based studies tended to show slightly higher ratios compared to community and hospital-based studies (Supplementary Table S5).

The funnel plot shows a generally symmetrical distribution of studies (Supplementary Fig. 4). The Harbord regression test for small-study effects indicates no evidence of publication bias (*t* = 0.29, *P* = 0.78), meaning that the bias estimate is small and non-significant. The trim-and-fill procedure did not add any studies (0 imputed), further supporting the absence of funnel plot asymmetry. After adjustment, the pooled effect size remained almost unchanged (OR = 0.94; 95% CI 0.63–1.40).

### Comparison of diagnostic methods for *T. trichiura* infections

**Fig. 6:**
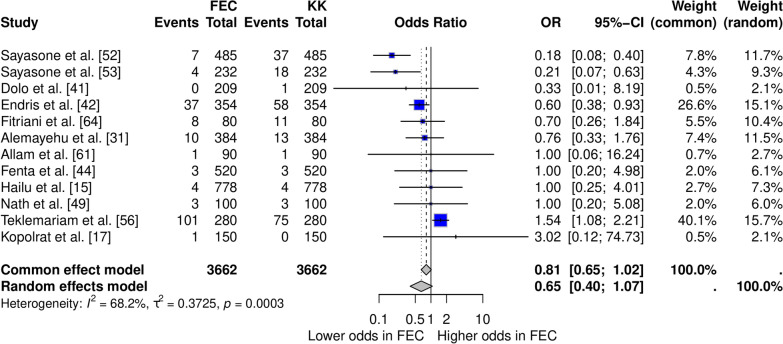
The Forest plot shows the pooled odds ratio for *Trichuris trichiura* infections comparing FEC with KK. Each horizontal line represents an individual study with its corresponding odds ratio (OR) and 95% confidence interval (CI). The blue squares indicate the point estimates of the ORs, and their sizes are proportional to the weight assigned to each study in the meta-analysis. The diamond at the bottom represents the pooled effect estimate for both the common (fixed) and random-effects model

The meta-analysis of the OR for *T. trichiura* infection included 12 studies comprising a total of 3662 stool samples across two diagnostic methods. Both the random-effects and fixed-effect models yielded nonsignificant pooled ORs of 0.65 (95% CI 0.40–1.07, *P* = 0.09, *I*^*2*^ = 68.2%) and 0.81 (95% CI 0.65–1.02, *P* = 0.08, Fig. 6), respectively.

The meta-regression analysis revealed that the continent (*P* = 0.0436) and participant type (*P* = 0.0283) significantly influenced the proportion ratio of *T. trichiura* detection between FEC and KK, while study design had no significant effect (*P* = 0.8323). Studies from Africa showed comparable detection rates between methods (OR = 0.94 in Ethiopia), whereas studies from Asia tended to favor KK, particularly in Lao PDR, where FEC yielded substantially lower detection (OR = 0.19, 95% CI 0.10–0.36, Supplementary Table S5).

The funnel plot appears largely symmetrical (Supplementary Fig. 5), and the Harbord regression test for small-study effects shows no evidence of publication bias (*t* = −0.52, *P* = 0.62), indicating that smaller studies do not systematically report stronger or weaker effects. The trim-and-fill analysis suggested that two studies might be missing on the left side of the funnel. After imputing these studies, the adjusted pooled effect size remained unchanged (OR = 0.94; 95% CI 0.51–1.72), confirming that any potential asymmetry does not meaningfully influence the overall result.

### Comparison of diagnostic methods for *S. stercoralis* infections

**Fig. 7:**
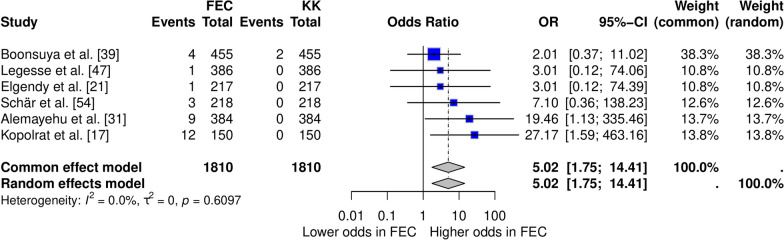
The Forest plot shows the pooled odds ratio for *Strongyloides stercoralis* infections comparing FEC with KK. Each horizontal line represents an individual study with its corresponding odds ratio (OR) and 95% confidence interval (CI). The blue squares indicate the point estimates of the ORs, and their sizes are proportional to the weight assigned to each study in the meta-analysis. The diamond at the bottom represents the pooled effect estimate for both the common (fixed) and random-effects model

The meta-analysis of the odds ratio (OR) for *S. stercoralis* infection included six studies comprising a total of 1810 stool samples analyzed using two diagnostic methods. Both the random-effects and fixed-effects models yielded a significant pooled OR of 5.02 (95% CI 1.75–14.41, *P* = 0.003, *I*^*2*^ = 0%, Fig. 7).

Owing to the limited number of studies included (six only), meta-regression and subgroup analyses could not be performed. In addition, the formal statistical test for small-study effects could not be conducted. Therefore, the funnel plot should be interpreted with caution, given its limited power to detect asymmetry. The trim-and-fill method suggested that one study might be missing; however, after imputing this study, the pooled effect size remained strong and statistically significant (OR = 3.83; 95% CI 1.43–10.30), indicating that any potential asymmetry does not materially change the overall conclusion.

### Comparison of diagnostic methods for *S. mansoni* infections

**Fig. 8:**
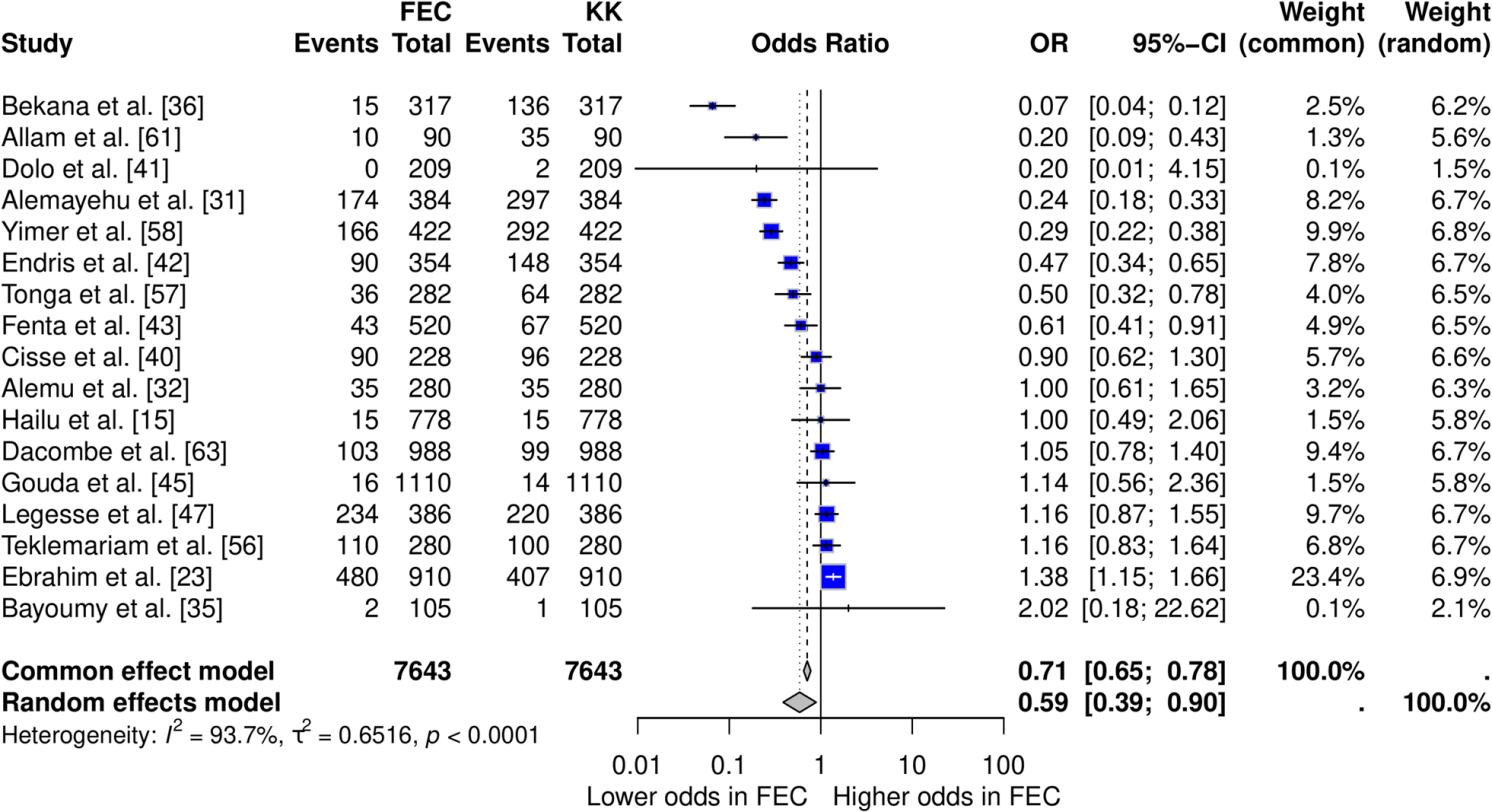
The Forest plot shows the pooled odds ratio for *Schistosoma mansoni* infections comparing FEC with KK. Each horizontal line represents an individual study with its corresponding odds ratio (OR) and 95% confidence interval (CI). The blue squares indicate the point estimates of the ORs, and their sizes are proportional to the weight assigned to each study in the meta-analysis. The diamond at the bottom represents the pooled effect estimate for both the common (fixed) and random-effects model

The meta-analysis of the OR for *S. mansoni* infection included 17 studies comprising a total of 7643 stool samples analyzed using two diagnostic methods. The random-effects model yielded a significant pooled OR of 0.59 (95% CI 0.39–0.90, *P* = 0.01, *I*^*2*^ = 93.7%), while the fixed-effect model produced a significant pooled OR of 0.71 (95% CI 0.65–0.78, *P* < 0.001, Fig. 8).

The meta-regression analysis indicated that the study design, country, and participant type did not significantly influence the pooled OR of *S. mansoni* detection between FEC and KK (*P* > 0.05). The detection ratio was lower in Ethiopia (OR = 0.50, 95% CI 0.27–0.92), while studies from Malawi reported similar performance between the two methods. Subgrouping by participant type revealed that schoolchildren showed significantly lower detection by FEC (OR = 0.49, 95% CI 0.28–0.84) compared with other groups (Supplementary Table S5).

The funnel plot appears mostly symmetrical (Supplementary Fig. 7), and the Harbord regression test shows no evidence of small-study effects (*t* = −0.74, *P* = 0.47). The bias estimate is small and non-significant, suggesting a low likelihood of publication bias. The trim-and-fill procedure suggested that two studies might be missing. After imputing these studies, the pooled effect size (OR = 0.75; 95% CI 0.45–1.23) changed only slightly and remained statistically nonsignificant. This suggests that any potential asymmetry does not significantly impact the overall conclusion.

### Comparison of diagnostic methods for *O. viverrini* infections

**Fig. 9:**
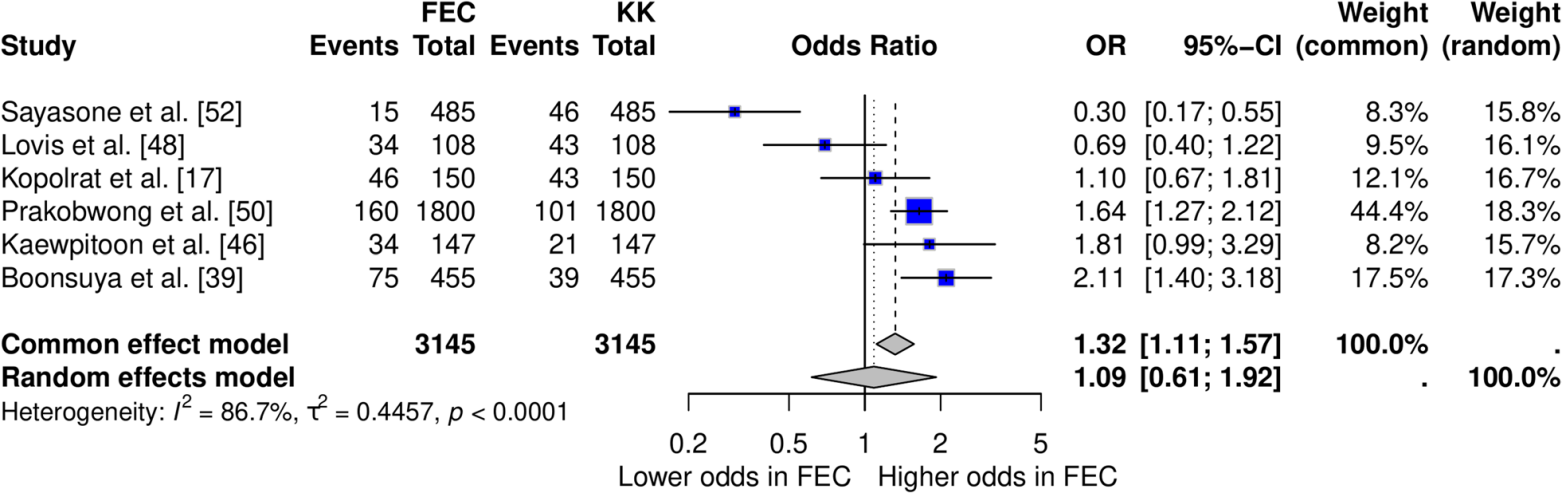
The Forest plot shows the pooled odds ratio for *Opisthorchis viverrini* infections comparing FEC with KK. Each horizontal line represents an individual study with its corresponding odds ratio (OR) and 95% confidence interval (CI). The blue squares indicate the point estimates of the ORs, and their sizes are proportional to the weight assigned to each study in the meta-analysis. The diamond at the bottom represents the pooled effect estimate for both the common (fixed) and random-effects model

The meta-analysis of the OR for *O. viverrini* infection included six studies comprising a total of 3145 stool samples analyzed using two diagnostic methods. The random-effects model yielded a nonsignificant pooled OR of 1.09 (95% CI 0.61–1.92, *P* = 0.78, *I*^*2*^ = 86.7%), while the fixed-effect model produced a significant pooled OR of 1.32 (95% CI 1.11–1.57, *P* = 0.002, Fig. 9).

The subgroup analysis of *O. viverrini* detection showed that the KK detected significantly higher proportions of *O. viverrini* infections in Thailand (OR = 1.65, 95% CI 1.33–2.03), whereas the detection rate was lower in Lao PDR (0.46, 95% CI 0.21–1.04). By participant type, no significant differences were found (*P* = 0.1826), though detection appeared slightly higher among migrant workers (OR = 1.81) compared with community participants (OR = 0.98, Supplementary Table S5).

Owing to the limited number of studies included (six only), meta-regression and subgroup analyses could not be performed. In addition, the formal statistical test for small-study effects could not be conducted. Therefore, the funnel plot should be interpreted with caution, given its limited power to detect asymmetry. The trim-and-fill method suggested that one study might be missing; however, after imputing this study, the pooled effect size remained strong and statistically significant (OR = 1.41; 95% CI 0.69–2.89), indicating that any potential asymmetry does not materially change the overall conclusion.

## Discussion

This systematic review and meta-analysis compared the performance of FEC with the single-slide KK across intestinal helminths, including both STH and non-STH species. Meta-analysis findings from 40 studies revealed no consistent superiority of FEC or KK when all helminths were considered together. Nevertheless, the presence of high heterogeneity affects the conclusion of this finding, as the fixed-effects model indicated slightly higher odds of detection by KK. This heterogeneity may contribute to differences in study designs, geographical distribution, and participants included in the studies. Further subgroup analyses demonstrated notable differences by region and participant type. For example, higher ORs favoring concentration methods were observed in Thailand and Cambodia, whereas studies from Ethiopia and the Philippines tended to favor KK. These findings underscore that diagnostic performance is context-dependent and may reflect differences in laboratory expertise, species distribution, and patterns of infection intensity across endemic areas.

When the meta-analysis focused on STHs, the results also showed no overall superiority of either technique for detecting combined STH infections. Subgroup patterns were similar to the overall helminth results, with diagnostic accuracy studies generally showing stronger performance of FEC compared with cross-sectional studies. This may reflect more rigorous laboratory procedures or more controlled sample processing environments, which are typically found in diagnostic accuracy studies.

For hookworm infections, the meta-analysis showed no significant difference between FEC and KK, although fixed-effect analyses favored the latter. The absence of publication bias and the minimal impact of trim-and-fill adjustments increase confidence in these conclusions. Geographic differences were pronounced in this analysis: studies in Asia consistently showed lower detection rates by concentration methods, whereas African studies showed comparable or slightly higher detection rates. Hookworm eggs are sensitive to clearing and glycerol effects during KK slide preparation [9]; therefore, the results of slide reading on hookworm may be missed or negative in case of delays between the slide preparation and reading. Furthermore, varying the time between the stool sample collection and the laboratory analysis may decrease hookworm fecal egg counts [10]. Delays in sample collection and transport to the laboratory may result in a negative hookworm detection owing to decreased hookworm retrieval from stools [10]. Lower hookworm detection by FEC may also be explained by the fact that some ova will not be deposited during the sedimentation step [10]. There were recommendations to increase the sensitivity of KK by performing multiple KK smears per sample, as only a small number of stool samples were used during the sample preparation [5, 7].

The detection of *A. lumbricoides* showed comparable performance between the two diagnostic methods, with no significant differences under either effect models. Meta-regression, however, identified the country and continent as significant moderators, suggesting that regional operational factors play a role. The subgroup analyses showed that African studies tended to report higher detection with FEC, whereas several Asian studies favored KK. These patterns may relate to egg morphology and density, as *Ascaris* eggs are relatively robust and may be efficiently recovered by concentration procedures.

Similarly, for *T. trichiura*, no significant differences were observed overall, although regional variability was again notable. Concentration methods performed poorly in some Asian settings, especially in the Lao PDR, whereas African studies reported more comparable sensitivity. Meta-regression findings that both continent and participant type influence detection suggest that stool characteristics and infection intensity, which vary between children, adults, and community samples, may affect diagnostic performance for this species.

In contrast to the STH findings, *S. stercoralis* detection clearly favored FEC, with a pooled OR exceeding 5.0. Given that KK is not recommended for *S. stercoralis* detection due to the fragile nature of the larvae and the filtering effect of the thick smear preparation, this result is expected and reinforces current diagnostic guidance. Although the small number of available studies limits generalizability, the large effect size and absence of heterogeneity suggest a robust finding.

For *S. mansoni*, the analysis revealed lower detection rates with concentration methods compared with KK. This aligns with the known advantages of thick smear methods for detecting *Schistosoma* eggs, particularly in moderate-to-high transmission settings where egg loads are higher. Subgroup analyses suggested that the difference was more pronounced in schoolchildren, possibly owing to age-related variations in infection intensity; schoolchildren may have similar water-contact behavior [31]. Previous studies have shown that the KK detects a higher proportion of *S. mansoni* infections than the FEC method, particularly at very low infection intensities (e.g., 36 EPG), which may not be detected by FEC [60]. However, these results remain controversial. More recent evidence indicates that sedimentation-based methods, such as FEC, may demonstrate higher sensitivity than KK, even when examining a single slide, potentially due to differences in analytical recovery and sample processing efficiency [65]. These contrasting findings highlight the need for cautious interpretation and suggest that diagnostic performance may vary depending on methodological and operational factors.

Performing multiple KK smears has been reported to increase sensitivity for *S. mansoni* detection [23, 48]. The higher sensitivity of the KK method may be attributed to the clearing of fibrous and coarse materials on the slide, allowing better visualization and recovery of eggs compared with sedimentation methods [66]. Furthermore, previous research found that both KK and FEC had approximately threefold higher sensitivity than direct wet-mount examination for detecting *S. mansoni* [32]. It has been suggested that KK is the preferred method in communities with known *S. mansoni* transmission, whereas in areas where KK may miss positive cases, combining KK with sedimentation methods is recommended [23]. Nevertheless, FEC was able to detect *S. mekongi* infections that KK missed [52]. Although the trim-and-fill procedure suggested the possibility of missing studies, the adjusted estimates remained consistent with the primary findings.

Although FEC has been reported to perform well for detecting helminthiasis, particularly in patients infected with *O. viverrini* [37, 61], the meta-analysis revealed no overall significant difference between concentration methods and KK. However, notable country-specific patterns were observed. Studies from Thailand, where the prevalence and intensity of *O. viverrini* infections tend to be higher, showed significantly higher detection rates with the KK. In contrast, FEC performed more favorably in studies conducted in the Lao PDR. These discrepancies may be related to the small size and dense nature of *O. viverrini* eggs, which can be lost during sedimentation in some concentration procedures but are more easily visualized on thick smear slides. In addition, several reports have suggested that both concentration methods and KK failed to differentiate between minute intestinal fluke (MIF) eggs and *O. viverrini* eggs [67, 68].

The declining prevalence of STHs and schistosomiasis in many settings, driven by the success of MDA programs, highlights the need for highly sensitive diagnostics. Both the KK and FEC suffer from reduced sensitivity in low-intensity infections, although the latter may offer advantages by processing larger stool volumes [5]. The use of multiple KK smears or a combination of methods is recommended in such settings to improve detection [52]. Beyond diagnostic accuracy, the choice between KK and FEC often depends on operational feasibility. The KK method is favored in resource-limited field settings owing to its low cost, simplicity, and lack of electricity or expensive equipment requirements, such as centrifuges [13, 69]. However, a major logistical limitation of KK is the need for rapid examination, particularly for hookworm eggs, which can over-clear and become invisible within hours of slide preparation [10]. In contrast, FEC enables the preservation of stool samples, allowing for batch processing and delayed examination without significant loss of morphology [42]. This makes FEC advantageous for surveys where immediate microscopy is not feasible. Furthermore, FEC offers a broader diagnostic spectrum, capable of detecting intestinal protozoan cysts and trophozoites that are missed by KK [13].

The present systematic review and meta-analysis acknowledge a few limitations. First, heterogeneity was consistently high. This reflects differences in stool preservation, slide preparation, technician expertise, time between sample collection and examination, and laboratory protocols, all of which can profoundly affect diagnostic outputs. Despite this variability, publication bias was not evident across the main analyses, and trim-and-fill adjustments had minimal impact, strengthening confidence in the observed patterns. The findings from the systematic review and meta-analysis emphasize that no single diagnostic method is universally superior for all intestinal helminths. The choice of techniques should consider the target species, local epidemiology, stool characteristics, and the availability of laboratory expertise. KK remains valuable for detecting STHs and schistosomes in moderate-to-high-intensity settings, whereas concentration methods may be preferable for targeting *S. stercoralis* or when screening for a broader range of helminths.

## Conclusions

FEC and the Kato–Katz thick smear demonstrate comparable performance in detecting most intestinal helminths, with species and region-specific differences. FEC is markedly superior for detecting *S. stercoralis*, whereas Kato–Katz thick smear performs better for *S. mansoni* and may detect higher proportions of *O. viverrini* in certain settings. Future research using standardized protocols, multi-slide examination, and integrated diagnostic approaches is needed to refine diagnostic recommendations tailored to specific epidemiological contexts.

## Supplementary Information

Additional file 1.

Additional file 2.

Additional file 3.

Additional file 4.

Additional file 5.

Additional file 6.

Additional file 7.

Additional file 8.

Additional file 9.

Additional file 10.

Additional file 11.

## Data Availability

All data related to the present study are available in this manuscript and in the Supplementary Table S1, Supplementary Table S2, Supplementary Table S3, and Supplementary Table S4 files.
