# Supplementary figures and images for: Comparison of Ritchie and Kato–Katz methods for the detection of intestinal helminths in humans: a systematic review and meta-analysis
Source: Parasit Vectors. 2026 May 15;19:282. doi: 10.1186/s13071-026-07437-7 (PMC13344047; doi:10.1186/s13071-026-07437-7)

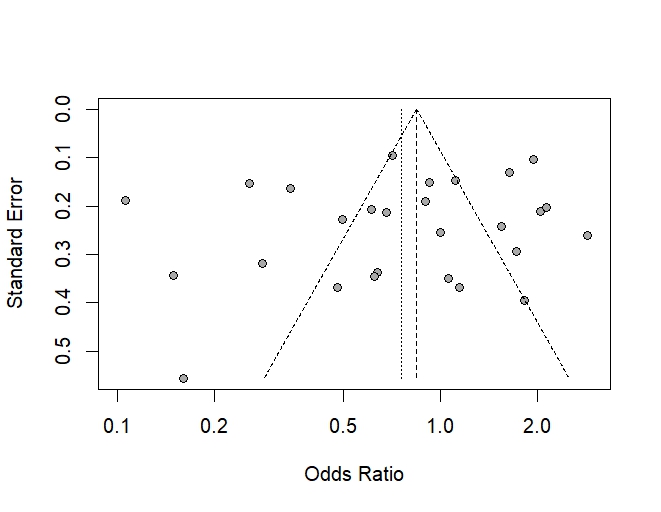

Supplement: Supplementary file 1 — Additional file 1. [file 13071_2026_7437_MOESM1_ESM.jpeg]

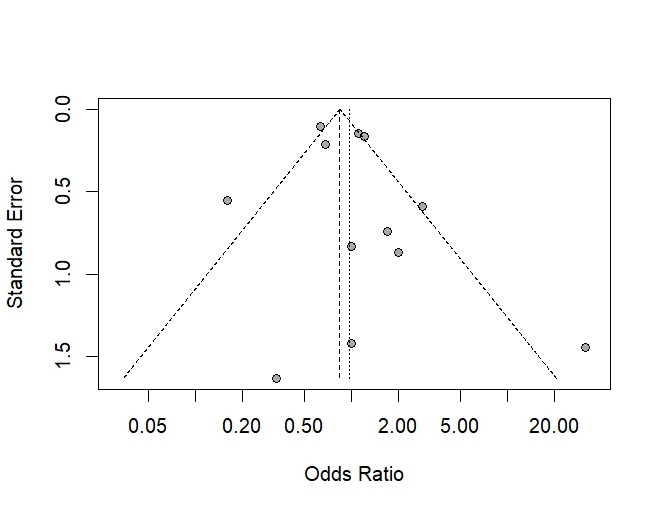

Supplement: Supplementary file 2 — Additional file 2. [file 13071_2026_7437_MOESM2_ESM.jpeg]

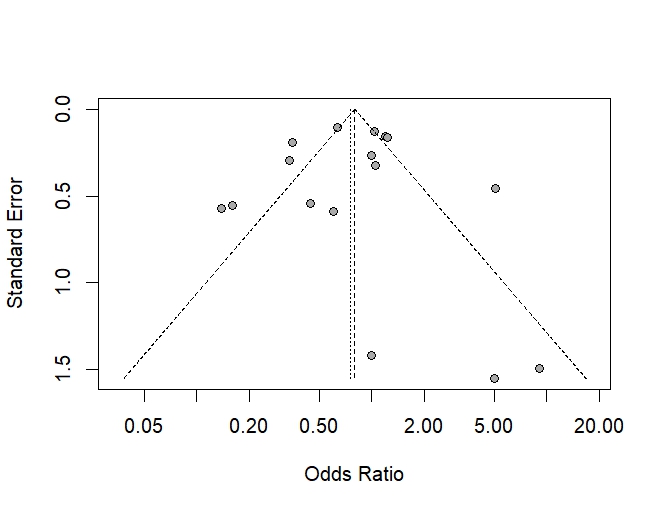

Supplement: Supplementary file 3 — Additional file 3. [file 13071_2026_7437_MOESM3_ESM.jpeg]

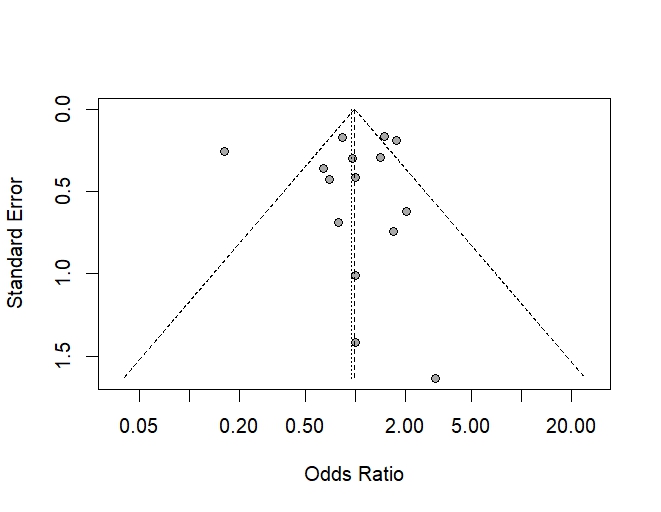

Supplement: Supplementary file 4 — Additional file 4. [file 13071_2026_7437_MOESM4_ESM.jpeg]

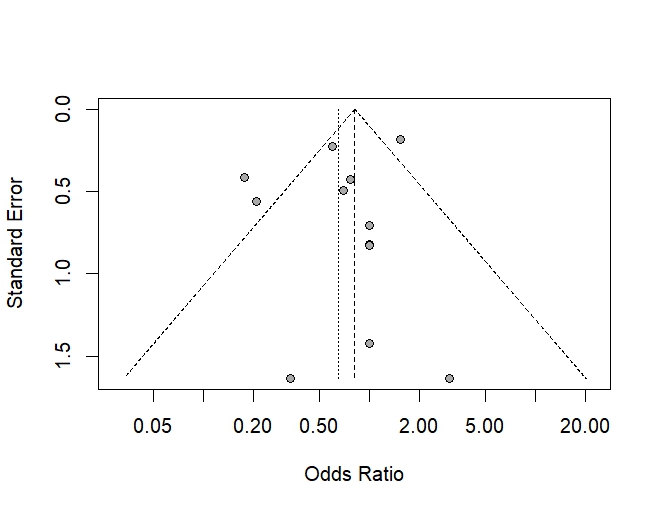

Supplement: Supplementary file 5 — Additional file 5. [file 13071_2026_7437_MOESM5_ESM.jpeg]

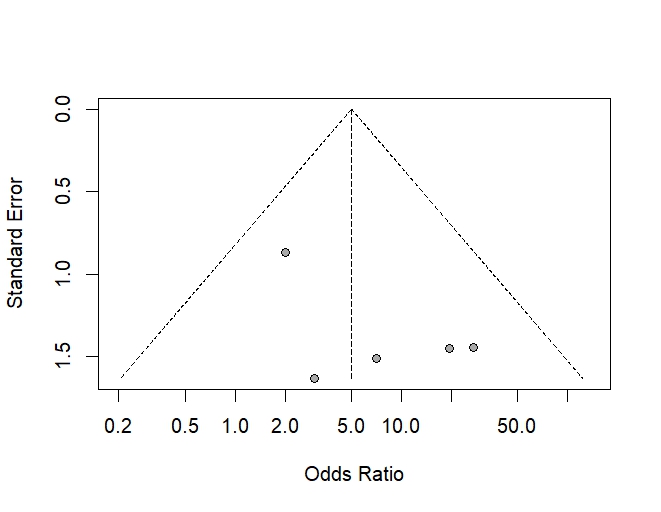

Supplement: Supplementary file 6 — Additional file 6. [file 13071_2026_7437_MOESM6_ESM.jpeg]

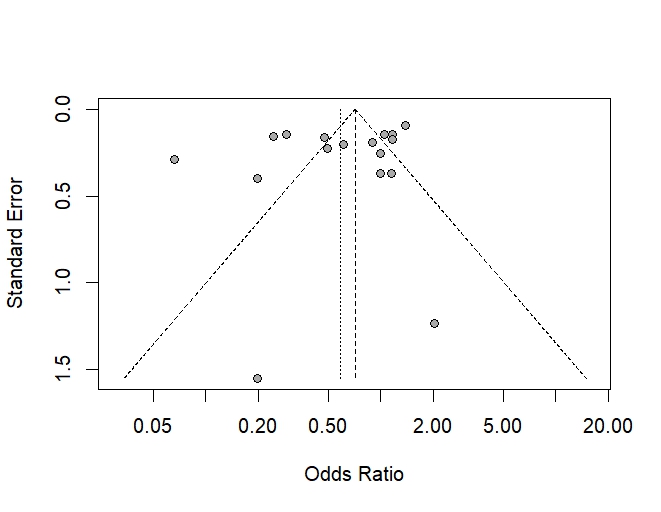

Supplement: Supplementary file 7 — Additional file 7. [file 13071_2026_7437_MOESM7_ESM.jpeg]
