# Supplementary material for: Comparison of Ritchie and Kato–Katz methods for the detection of intestinal helminths in humans: a systematic review and meta-analysis
Source: Parasit Vectors. 2026 May 15;19:282. doi: 10.1186/s13071-026-07437-7 (PMC13344047; doi:10.1186/s13071-026-07437-7)
Supplement: Supplementary file 8 — Additional file 8. [file 13071_2026_7437_MOESM8_ESM.docx]

**Table S1. Search terms**

**General keywords**

(“Thick smear” OR “Kato-Katz” OR “Kato smear” OR “Kato’s thick smear” OR “Kato-thick smear”) AND (“Formol–ether Concentration” OR “Formol ether sedimentation” OR “Formalin–ether Concentration” OR “Formalin ether Concentration” OR “Formalin ethyl acetate concentration” OR “Formalin–ethyl acetate concentration” OR “Formalin ethyl acetate sedimentation” OR “Formalin–ethyl acetate sedimentation”)

PubMed 9 October 2025

| No. | Key concept | Search terms | Results |
| --- | --- | --- | --- |
| 1. | Thick smear | “Thick smear”[MeSH Terms] OR “Kato-Katz”[MeSH Terms] OR “Kato smear”[MeSH Terms] OR “Kato’s thick smear”[MeSH Terms] OR “Kato-thick smear”[MeSH Terms] OR “Thick smear”[Text Word] OR “Kato-Katz”[Text Word] OR “Kato smear”[Text Word] OR “Kato’s thick smear”[Text Word] OR “Kato-thick smear”[Text Word] | 2,158 |
| 2. | Formalin/Formal Concentration method | “Formol–ether Concentration”[MeSH Terms] OR “Formol ether sedimentation”[MeSH Terms] OR “Formalin–ether Concentration”[MeSH Terms] OR “Formalin ether Concentration”[MeSH Terms] OR “Formalin ethyl acetate concentration”[MeSH Terms] OR “Formalin–ethyl acetate concentration”[MeSH Terms] OR “Formalin ethyl acetate sedimentation”[MeSH Terms] OR “Formalin–ethyl acetate sedimentation”[MeSH Terms] OR “Formol–ether Concentration”[Text Word] OR “Formol ether sedimentation”[Text Word] OR “Formalin–ether Concentration”[Text Word] OR “Formalin ether Concentration”[Text Word] OR “Formalin ethyl acetate concentration”[Text Word] OR “Formalin–ethyl acetate concentration”[Text Word] OR “Formalin ethyl acetate sedimentation”[Text Word] OR “Formalin–ethyl acetate sedimentation”[Text Word] | 753 |
| 3. | 1 AND 2 | #1 AND #2 | 119 |

Embase 20 March 2025

| No. | Key concept | Search terms | Results |
| --- | --- | --- | --- |
| 1. | Thick smear | 'thick smear' OR 'kato–katz' OR 'kato smear' OR 'kato thick smear' OR 'kato–thick smear' OR 'thick smear':ti,ab,kw,de OR 'kato–katz':ti,ab,kw,de OR 'kato smear':ti,ab,kw,de OR 'kato s thick smear':ti,ab,kw,de OR 'kato-thick smear':ti,ab,kw,de | 2,966 |
| 2. | Formalin/Formal Concentration method | 'formol–ether concentration' OR 'formol ether sedimentation' OR 'formalin–ether concentration' OR 'formalin ether concentration' OR 'formalin ethyl acetate concentration' OR 'formalin–ethyl acetate concentration' OR 'formalin ethyl acetate sedimentation' OR 'formalin–ethyl acetate sedimentation' OR 'formol–ether concentration':ti,ab,kw,de OR 'formol ether sedimentation':ti,ab,kw,de OR 'formalin–ether concentration':ti,ab,kw,de OR 'formalin ether concentration':ti,ab,kw,de OR 'formalin ethyl acetate concentration':ti,ab,kw,de OR 'formalin–ethyl acetate concentration':ti,ab,kw,de OR 'formalin ethyl acetate sedimentation':ti,ab,kw,de OR 'formalin–ethyl acetate sedimentation':ti,ab,kw,de | 953 |
| 3. | #1 AND #2 | (*Cryptosporidium*/exp OR “*Cryptosporidium parvum”*/exp OR Coccidia/exp OR Cryptosporidiidae/exp OR Cryptosporidiosis/exp OR *Cryptosporidium*:ti,ab,kw,de OR “*Cryptosporidium parvum”*:ti,ab,kw,de OR Coccidia:ti,ab,kw,de OR Cryptosporidiidae:ti,ab,kw,de OR Cryptosporidiosis:ti,ab,kw,de) AND (Thailand:ti,ab,kw,de OR Thailand/exp OR Siam:ti,ab,kw,de OR Siam/exp) | 151 |

Scopus 9 October 2025

| No. | Key concept | Search terms | Results |
| --- | --- | --- | --- |
| 1. | Thick smear | TITLE-ABS-KEY ( "Thick smear" OR "Kato-Katz" OR "Kato smear" OR "Kato’s thick smear" OR "Kato-thick smear" ) | 2,640 |
| 2. | Formalin/Formal Concentration method | TITLE-ABS-KEY ( "Formol–ether Concentration" OR "Formol ether sedimentation" OR "Formalin–ether Concentration" OR "Formalin ether Concentration" OR "Formalin ethyl acetate concentration" OR "Formalin–ethyl acetate concentration" OR "Formalin ethyl acetate sedimentation" OR "Formalin–ethyl acetate sedimentation" ) | 945 |
| 3. | 1 AND 2 | ( TITLE-ABS-KEY ( "Thick smear" OR "Kato-Katz" OR "Kato smear" OR "Kato’s thick smear" OR "Kato-thick smear" ) ) AND ( TITLE-ABS-KEY ( "Formol–ether Concentration" OR "Formol ether sedimentation" OR "Formalin–ether Concentration" OR "Formalin ether Concentration" OR "Formalin ethyl acetate concentration" OR "Formalin–ethyl acetate concentration" OR "Formalin ethyl acetate sedimentation" OR "Formalin–ethyl acetate sedimentation" ) ) | 138 |

Journal@Ovid 9 October 2025

| No. | Key concept | Search terms | Results |
| --- | --- | --- | --- |
| 1. | Thick smear AND Formalin/Formal Concentration method | (“Thick smear” OR “Kato-Katz” OR “Kato smear” OR “Kato’s thick smear” OR “Kato-thick smear”) AND (“Formol–ether Concentration” OR “Formol ether sedimentation” OR “Formalin–ether Concentration” OR “Formalin ether Concentration” OR “Formalin ethyl acetate concentration” OR “Formalin–ethyl acetate concentration” OR “Formalin ethyl acetate sedimentation” OR “Formalin–ethyl acetate sedimentation”) | 18 |

Nursing & Allied Health Premium 9 October 2025

| No. | Key concept | Search terms | Results |
| --- | --- | --- | --- |
| 1. | Thick smear AND Formalin/Formal Concentration method | (“Thick smear” OR “Kato-Katz” OR “Kato smear” OR “Kato’s thick smear” OR “Kato-thick smear”) AND (“Formol–ether Concentration” OR “Formol ether sedimentation” OR “Formalin–ether Concentration” OR “Formalin ether Concentration” OR “Formalin ethyl acetate concentration” OR “Formalin–ethyl acetate concentration” OR “Formalin ethyl acetate sedimentation” OR “Formalin–ethyl acetate sedimentation”) | 84 |

Web of Science 10 October 2025

| No. | Key concept | Search terms | Results |
| --- | --- | --- | --- |
| 1. | Thick smear AND Formalin/Formal Concentration method | (“Thick smear” OR “Kato-Katz” OR “Kato smear” OR “Kato’s thick smear” OR “Kato-thick smear”) AND (“Formol–ether Concentration” OR “Formol ether sedimentation” OR “Formalin–ether Concentration” OR “Formalin ether Concentration” OR “Formalin ethyl acetate concentration” OR “Formalin–ethyl acetate concentration” OR “Formalin ethyl acetate sedimentation” OR “Formalin–ethyl acetate sedimentation”) | 113 |
