# Supplementary material for: Comparison of Ritchie and Kato–Katz methods for the detection of intestinal helminths in humans: a systematic review and meta-analysis
Source: Parasit Vectors. 2026 May 15;19:282. doi: 10.1186/s13071-026-07437-7 (PMC13344047; doi:10.1186/s13071-026-07437-7)
Supplement: Supplementary file 11 — Additional file 11. [file 13071_2026_7437_MOESM11_ESM.docx]

**Table S4. Meta-regression and subgroup analysis** **of the pooled proportion ratio of helminth proportion between concentration methods and Kato thick smear**

**1. Meta-regression analysis of the pooled proportion ratio of helminth proportion between concentration methods and Kato thick smear**

| **Covariates** | **tau^2^** | **Residual heterogeneity *I^2^* (%)** | **H^2^** | **R^2^** | **Test of moderators, *P* value** | **Number of studies** |
| --- | --- | --- | --- | --- | --- | --- |
| Study design | 0.69 | 94.19 | 17.20 | 0.00 | 0.40 | 27 |
| Country | 0.61 | 93.55 | 15.51 | 11.21 | 0.20 | 27 |
| Continent | 0.70 | 94.70 | 18.88 | 0.00 | 0.54 | 27 |
| Participants | 0.72 | 94.52 | 18.23 | 0.00 | 0.59 | 27 |

**2. Subgroup analysis of the pooled proportion ratio of helminth proportion between concentration methods and Kato thick smear**

| **Pooled prevalence** | **Subgroup** | **Test for subgroup difference** | **Proportion ratio (%)** [**95% CI**] | ***I^2^* (%)** | **Number of studies (subsets)** |
| --- | --- | --- | --- | --- | --- |
| Overall |  |  | 0.76 [0.55; 1.05] | 94.2 | 27 |
| Study designs |  | 0.22 |  |  |  |
|  | Cross-sectional study |  | 0.80 [0.56; 1.14] | 94.6 | 24 |
|  | Diagnostic accuracy study |  | 0.53 [0.30; 0.92] | 74.4 | 3 |
| Continent |  | 0.50 |  |  |  |
| Africa |  |  | 0.67 [0.44; 1.03] | 95.2 | 17 |
|  | Ethiopia |  | 0.51 [0.27; 0.97] | 97.2 | 9 |
|  | Egypt |  | 1.13 [0.51; 2.47] | 88.0 | 5 |
|  | Burkina Faso |  | 0.90 [0.62; 1.30] | N/A | 1 |
|  | Malawi |  | 0.71 [0.59; 0.86] | N/A | 1 |
|  | Cameroon |  | 0.50 [0.39; 0.78] | N/A | 1 |
| Asia |  |  | 1.01 [0.54; 1.91] | 89.4 | 8 |
|  | Thailand |  | 1.7314 [1.4369; 2.0864] | 0.0 | 4 |
|  | Philippines |  | 0.27 [0.08; 0.84] | 81.6 | 2 |
|  | Bangladesh |  | 0.62 [0.32; 1.23] | N/A | 1 |
|  | Cambodia |  | 2.04 [1.35; 3.08] | N/A | 1 |
| South America |  |  | 0.67 [0.47; 0.95] | 0.0 | 2 |
|  | Ecuador |  | 0.67 [0.47; 0.95] | 0.0 | 2 |
| Participants |  | 0.003 |  |  |  |
|  | Schoolchildren |  | 0.56 [0.34; 0.94] | 96.4 | 13 |
|  | Participants in community |  | 1.09 [0.57; 2.08] | 86.8 | 7 |
|  | Patients in hospital/health institution |  | 1.16 [0.81; 1.66] | 0.0 | 3 |
|  | Not specified |  | 0.71 [0.59; 0.86] | N/A | 1 |
|  | Migrant workers |  | 1.72 [0.97; 3.06] | N/A | 1 |
|  | Schoolchildren and adults |  | 0.62 [0.32; 1.23] | N/A | 1 |
|  | Pregnant women |  | 0.50 [0.32; 0.78] | N/A | 1 |

N/A, not assessed

**3. Meta-regression analysis of the pooled proportion ratio of soil-transmitted helminths proportion between concentration methods and Kato thick smear**

| **Covariates** | **tau^2^** | **Residual heterogeneity *I^2^* (%)** | **H^2^** | **R^2^** | **Test of moderators, *P* value** | **Number of studies** |
| --- | --- | --- | --- | --- | --- | --- |
| Study design | 0.44 | 78.56 | 4.66 | 0.00 | 0.53 | 12 |
| Country | 0.59 | 83.44 | 6.04 | 0.00 | 0.47 | 12 |
| Continent | 0.42 | 84.80 | 6.58 | 0.00 | 0.24 | 12 |
| Participants | 0.94 | 72.68 | 3.66 | 0.00 | 0.98 | 12 |

**4. Subgroup analysis of the pooled proportion ratio of soil-transmitted helminths proportion between concentration methods and Kato thick smear**

| **Pooled prevalence** | **Subgroup** | **Test for subgroup difference** | **Proportion ratio (%)** [**95% CI**] | ***I^2^* (%)** | **Number of studies (subsets)** |
| --- | --- | --- | --- | --- | --- |
| Overall |  |  | 0.98 [0.61; 1.55] | 71.3 | 12 |
| Study designs |  | 0.11 |  |  |  |
|  | Cross-sectional study |  | 1.09 [0.59; 2.03] | 66.0 | 10 |
|  | Diagnostic accuracy study |  | 0.64 [0.52; 0.79] | 0.0 | 2 |
| Continent |  | 0.19 |  |  |  |
| Africa |  |  | 0.89 [0.51; 1.57] | 76.6 | 8 |
|  | Ethiopia |  | 0.67 [0.20; 2.18] | 83.9 | 3 |
|  | Egypt |  | 1.91 [0.86; 4.21] | 0.0 | 3 |
|  | Malawi |  | 0.64 [0.52; 0.79] | N/A | 1 |
|  | Mali |  | 0.33 [0.01; 8.19] | N/A | 1 |
| Asia |  |  | 3.46 [0.59; 20.3] | 42.8 | 3 |
|  | Thailand |  | 3.46 [0.59; 20.3] | 42.8 | 3 |
| South America |  |  | 0.68 [0.45; 1.03] | N/A | 1 |
|  | Ecuador |  | 0.68 [0.45; 1.03] | N/A | 1 |
| Participants |  | 0.003 |  |  |  |
|  | Participants in community |  | 1.16[0.31; 4.26] | 76.5 | 6 |
|  | Schoolchildren |  | 1.11 [0.84; 1.47] | 0.0 | 2 |
|  | Patients in hospital/health institution |  | 1.24 [0.91; 1.70] | 0.0 | 2 |
|  | Not specified |  | 0.64 [0.52; 0.79] | N/A | 1 |
|  | Migrant workers |  | 1.00 [0.06; 16.14] | N/A | 1 |

N/A, not assessed

**5. Meta-regression analysis of the pooled proportion ratio of hookworm proportion between concentration methods and Kato thick smear**

| **Covariates** | **tau^2^** | **Residual heterogeneity *I^2^* (%)** | **H^2^** | **R^2^** | **Test of moderators, *P* value** | **Number of studies** |
| --- | --- | --- | --- | --- | --- | --- |
| Study design | 0.6120 | 89.15 | 9.22 | 0.99 | 0.1729 | 16 |
| Country | 0.4158 | 87.73 | 8.15 | 32.74 | 0.1532 | 16 |
| Continent | 0.2576 | 81.13 | 5.30 | 58.32 | 0.0017 | 16 |
| Participants | 0.6924 | 90.28 | 10.29 | 0.00 | 0.6721 | 16 |

**6. Subgroup analysis of the pooled proportion ratio of hookworm proportion between concentration methods and Kato thick smear**

| **Pooled prevalence** | **Subgroup** | **Test for subgroup difference** | **Proportion ratio (%)** [**95% CI**] | ***I^2^* (%)** | **Number of studies (subsets)** |
| --- | --- | --- | --- | --- | --- |
| Overall |  |  | 0.7508 [0.4765; 1.1828] | 83.2 | 16 |
| Study designs |  | 0.2318 |  |  |  |
|  | Cross-sectional study |  | 0.8513 [0.5288; 1.3704] | 82.1 | 14 |
|  | Diagnostic accuracy study |  | 0.3297 [0.0750; 1.4485] | 85.5 | 2 |
| Continent |  | 0.0001 |  |  |  |
| Africa |  |  | 1.0966 [0.6632; 1.8133] | 80.4 | 10 |
|  | Ethiopia |  | 1.1530 [0.6394; 2.0792] | 73.2 | 8 |
|  | Egypt |  | 5.0464 [0.2409; 105.7288] | N/A | 1 |
|  | Malawi |  | 0.6373 [0.5161; 0.7870] | N/A | 1 |
|  | Mali |  |  |  |  |
| Asia |  |  | 0.3495 [0.2636; 0.4633] | 0.0 | 6 |
|  | Lao PDR |  | 0.3629 [0.2545; 0.5175] | 0.0 | 2 |
|  | Thailand |  | 1.0000 [0.0620; 16.1397] | N/A | 1 |
|  | Indonesia |  | 0.1388 [0.0453; 0.4248] | N/A | 1 |
|  | Bangladesh |  | 0.6053 [0.1910; 1.9183] | N/A | 1 |
|  | Cambodia |  | 0.3365 [0.1881; 0.6021] | N/A | 1 |
| Participants |  | 0.0883 |  |  |  |
|  | Schoolchildren |  | 1.1433 [0.5234; 2.4974] | 77.8 | 7 |
|  | Participants in community |  | 0.3310 [0.2096; 0.5229] | 41.3 | 4 |
|  | Patients in hospital/health institution |  | 0.6598 [0.1957; 2.2247] | 95.9 | 2 |
|  | Not specified |  | 0.6373 [0.5161; 0.7870] | N/A | 1 |
|  | Migrant workers |  | 1.0000 [0.0620; 16.1397] | N/A | 1 |
|  | Schoolchildren and adults |  | 0.6053 [0.1910; 1.9183] | N/A | 1 |

N/A, not assessed

**7. Meta-regression analysis of the pooled proportion ratio of *Ascaris* proportion between concentration methods and Kato thick smear**

| **Covariates** | **tau^2^** | **Residual heterogeneity *I^2^* (%)** | **H^2^** | **R^2^** | **Test of moderators, *P* value** | **Number of studies** |
| --- | --- | --- | --- | --- | --- | --- |
| Study design | 0.4003 | 80.89 | 5.23 | 0.00 | 0.8999 | 15 |
| Country | 0.0709 | 46.77 | 1.88 | 81.31 | 0.0001 | 15 |
| Continent | 0.1581 | 60.52 | 2.53 | 58.34 | 0.0023 | 15 |
| Participants | 0.2501 | 72.63 | 3.65 | 34.10 | 0.2447 | 15 |

**8. Subgroup analysis of the pooled proportion ratio of *Ascaris* proportion between concentration methods and Kato thick smear**

| **Pooled prevalence** | **Subgroup** | **Test for subgroup difference** | **Proportion ratio (%)** [**95% CI**] | ***I^2^* (%)** | **Number of studies (subsets)** |
| --- | --- | --- | --- | --- | --- |
| Overall |  |  |  |  |  |
| Study designs |  | 0.8534 |  |  |  |
|  | Cross-sectional study |  | 0.9528 [0.6202; 1.4637] | 83.5 | 13 |
|  | Diagnostic accuracy study |  | 0.8511 [0.2784; 2.6017] | 0.0 | 2 |
| Continent |  | 0.0943 |  |  |  |
| Africa |  |  | 1.1926 [0.9215; 1.5435] | 41.6 | 10 |
|  | Ethiopia |  | 1.1535 [0.8716; 1.5265] | 58.5 | 7 |
|  | Egypt |  | 1.6853 [0.7243; 3.9213] | 0.0 | 3 |
| Asia |  |  | 0.5076 [0.1930; 1.3350] | 72.0 | 5 |
|  | Lao PDR |  | 0.2364 [0.0566; 0.9869] | 36.8 | 2 |
|  | Thailand |  | 3.0201 [0.1221; 74.7298] | N/A | 1 |
|  | Indonesia |  | 0.7895 [0.2041; 3.0543] | N/A | 1 |
|  | Bangladesh |  | 0.7004 [0.3045; 1.6108] | N/A | 1 |
| Participants |  | 0.6670 |  |  |  |
|  | Schoolchildren |  | 1.1377 [0.8707; 1.4866] | 46.2 | 9 |
|  | Participants in community |  | 0.7552 [0.1065; 5.3534] | 87.9 | 3 |
|  | Patients in hospital/health institution |  | 1.5160 [0.4170; 5.5114] | 0.0 | 2 |
|  | Schoolchildren and adults |  | 0.7004 [0.3045; 1.6108] | N/A | 1 |

N/A, not assessed

**9. Meta-regression analysis of the pooled proportion ratio of *Trichuris* proportion between concentration methods and Kato thick smear**

| **Covariates** | **tau^2^** | **Residual heterogeneity *I^2^* (%)** | **H^2^** | **R^2^** | **Test of moderators, *P* value** | **Number of studies** |
| --- | --- | --- | --- | --- | --- | --- |
| Study design | 0.4244 | 68.35 | 3.16 | 0.00 | 0.8323 | 12 |
| Country | N/A | N/A | N/A | N/A | N/A | N/A |
| Continent | 0.1999 | 48.87 | 1.96 | 46.33 | 0.0436 | 12 |
| Participants | 0.1374 | 42.63 | 1.74 | 63.12 | 0.0283 | 12 |

**10. Subgroup analysis of the pooled proportion ratio of *Trichuris* proportion between concentration methods and Kato thick smear**

| **Pooled prevalence** | **Subgroup** | **Test for subgroup difference** | **Proportion ratio (%)** [**95% CI**] | ***I^2^* (%)** | **Number of studies (subsets)** |
| --- | --- | --- | --- | --- | --- |
| Overall |  |  |  |  |  |
| Study designs |  | 0.8182 |  |  |  |
|  | Cross-sectional study |  | 0.6387 [0.3622; 1.1262] | 73.9 | 10 |
|  | Diagnostic accuracy study |  | 0.7246 [0.2902; 1.8093] | 0.0 | 2 |
| Continent |  | 0.0919 |  |  |  |
| Africa |  |  | [0.5712; 1.4799] | 48.7 | 7 |
|  | Ethiopia |  | 0.9370 [0.5698; 1.5410] | 64.4 | 5 |
|  | Egypt |  | 1.0000 [0.0616; 16.2377] | N/A | 1 |
|  | Mali |  | 0.3317 [0.0134; 8.1903] | N/A | 1 |
| Asia |  |  | [0.1779; 0.9247] | 54.4 | 5 |
|  | Lao PDR |  | 0.1879 [0.0975; 0.3622] | 0.0 | 2 |
|  | Thailand |  | 3.0201 [0.1221; 74.7298] | N/A | 1 |
|  | Indonesia |  | 0.6970 [0.2645; 1.8362] | N/A | 1 |
|  | Bangladesh |  | 1.0000 [0.1969; 5.0777] | N/A | 1 |

N/A, not assessed

**11. Meta-regression analysis of the pooled proportion ratio of *S. mansoni* proportion between concentration methods and Kato thick smear**

| **Covariates** | **tau^2^** | **Residual heterogeneity *I^2^* (%)** | **H^2^** | **R^2^** | **Test of moderators, *P* value** | **Number of studies** |
| --- | --- | --- | --- | --- | --- | --- |
| Study design | 0.7021 | 94.77 | 19.12 | 0.00 | 0.7457 | 17 |
| Country | 0.8403 | 95.07 | 20.29 | 0.00 | 0.8995 | 17 |
| Continent | N/A | N/A | N/A | N/A | N/A | N/A |
| Participants | 0.7458 | 94.07 | 16.87 | 0.00 | 0.7072 | 17 |

**12. Subgroup analysis of the pooled proportion ratio of *S. mansoni* proportion between concentration methods and Kato thick smear**

| **Pooled prevalence** | **Subgroup** | **Test for subgroup difference** | **Proportion ratio (%)** [**95% CI**] | ***I^2^* (%)** | **Number of studies (subsets)** |
| --- | --- | --- | --- | --- | --- |
| Overall |  |  | 0.5895 [0.3882; 0.8950] | 93.7 | 17 |
| Study designs |  | 0.7742 |  |  |  |
|  | Cross-sectional study |  | 0.6051 [0.3866; 0.9470] | 94.1 | 15 |
|  | Diagnostic accuracy study |  | 0.4721 [0.0919; 2.4242] | 93.5 | 2 |
| Continent |  |  |  |  |  |
| Africa |  | 0.0510 | 0.5895 [0.3882; 0.8950] | 93.7 | 17 |
|  | Ethiopia |  | 0.4963 [0.2685; 0.9174] | 94.8 | 9 |
|  | Egypt |  | 0.7996 [0.2833; 2.2573] | 86.8 | 4 |
|  | Burkina Faso |  | 0.8967 [0.6171; 1.3030] | N/A | 1 |
|  | Malawi |  | 1.0451 [0.7811; 1.3983] | N/A | 1 |
|  | Cameroon |  | 0.4985 [0.3187; 0.7796] | N/A | 1 |
|  | Mali |  | 0.1981 [0.0095; 4.1512] | N/A | 1 |
| Participants |  | 0.0194 |  |  |  |
|  | Schoolchildren |  | 0.4896 [0.2843; 0.8431] | 93.9 | 11 |
|  | Participants in community |  | 0.9703 [0.2241; 4.2016] | 35.8 | 2 |
|  | Patients in hospital/health institution |  | 1.0294 [0.6304; 1.6810] | 0.0 | 2 |
|  | Not specified |  | 1.0451 [0.7811; 1.3983] | N/A | 1 |
|  | Pregnant women |  | 0.4985 [0.3187; 0.7796] | N/A | 1 |

N/A, not assessed

**13. Subgroup analysis of the pooled proportion ratio of *O. viverrini* proportion between concentration methods and Kato thick smear**

| **Pooled prevalence** | **Subgroup** | **Test for subgroup difference** | **Proportion ratio (%)** [**95% CI**] | ***I^2^* (%)** | **Number of studies (subsets)** |
| --- | --- | --- | --- | --- | --- |
| Overall |  |  | 1.0863 [0.6135; 1.9233] | 86.7 | 6 |
| Study designs | Cross-sectional study | N/A | 1.0863 [0.6135; 1.9233] | 86.7 | 6 |
| Continent | Asia | N/A | 1.0863 [0.6135; 1.9233] | 86.7 | 6 |
| Country |  | 0.0029 |  |  |  |
|  | Thailand |  | 1.6452 [1.3323; 2.0317] | 25.0 | 4 |
|  | Lao PDR |  | 0.4631 [0.2065; 1.0386] | 74.3 | 2 |
| Participants |  | 0.1826 |  |  |  |
|  | Participants in community |  | 0.9846 [0.5093; 1.9034] | 89.0 | 5 |
|  | Migrant worker |  | 1.8053 [0.9905; 3.2906] | N/A | 1 |

N/A, not assessed
